# Trends and disparities in the US colorectal cancer mortality, 1999–2023: an analysis of the CDC WONDER database

**DOI:** 10.3389/fonc.2025.1693240

**Published:** 2025-12-17

**Authors:** Yingkai Feng, Xinyu Fa, Jianping Liu, Hong Qi

**Affiliations:** 1 Department of General Surgery, Qingdao Hospital, University of Health and Rehabilitation Sciences (Qingdao Municipal Hospital), Qingdao, Shandong, China; 2 Department of Health Care, First Section, Qingdao Hospital, University of Health and Rehabilitation Sciences (Qingdao Municipal Hospital), Qingdao, Shandong, China; 3 Department of Medical Affairs, Qingdao Hospital, University of Health and Rehabilitation Sciences (Qingdao Municipal Hospital), Qingdao, Shandong, China

**Keywords:** cancer mortality, colorectal cancer, disease progression, malignancy, population surveillance

## Abstract

**Background:**

Colorectal cancer (CRC) remains the second leading cause of cancer-related death in the USA. This study systematically assessed the spatiotemporal evolution of CRC mortality from 1999 to 2023 and explored sociodemographic and geographic disparities.

**Methods:**

Death-certificate data for adults aged ≥ 25 years were extracted from the CDC WONDER database. CRC deaths were identified using ICD-10 codes C18–C20. Age-adjusted mortality rates (AAMR) were calculated with the 2000 US standard population. Stratified analyses were performed by age, sex, race/ethnicity, census region, state, and National Center for Health Statistics urban–rural classification. Joinpoint regression estimated annual percent change (APC) and average annual percent change (AAPC).

**Results:**

AAMR rose annually by 0.45% in the 25–34-year group and 1.03% in the 35–44-year group but declined significantly among individuals ≥ 55 years (AAPC −1.43% to −3.20%). Adults ≥ 75 years accounted for more than half of CRC deaths. The South registered the highest AAMR (21.13 per 100,000), whereas the Northeast had the lowest (17.31 per 100,000). Non-Hispanic Black individuals experienced the highest AAMR (24.90 per 100,000). Nonmetropolitan counties showed higher AAMR (23.16 per 100,000) than metropolitan counties (18.69 per 100,000).

**Conclusion:**

Despite an overall decline in US CRC mortality, rising risk among young adults and pronounced disparities across regions, racial/ethnic groups, and urban–rural settings persist. Targeted screening and intervention strategies for younger populations, high-burden areas, and vulnerable groups are essential to accelerate equitable reductions in CRC mortality.

## Introduction

Colorectal cancer (CRC) is the fourth most frequently diagnosed malignancy and the second leading cause of cancer-related death in both men and women in the USA (1). Although substantial progress has been made in early detection and treatment, CRC continues to impose a major health burden. Over the past decades, population-level CRC age-adjusted mortality rates (AAMRs) have shown a downward trajectory, largely attributed to improvements in screening uptake, therapeutic innovation, and broader public health awareness (2). Since the early 2000s, the widespread adoption of colonoscopy (3), fecal immunochemical testing (FIT) (4), and multitarget stool DNA (5) testing has significantly improved early detection. In 2018, the American Cancer Society lowered the recommended screening age from 50 to 45 years for average-risk adults, a change adopted by the US Preventive Services Task Force in 2021, reflecting growing recognition of early-onset disease (6). Concurrently, therapeutic breakthroughs have collectively extended survival across disease stages, such as the establishment of folinic acid, fluorouracil (5-FU), and oxaliplatin (FOLFOX) as the adjuvant standard in the Multicenter International Study of Oxaliplatin/5-Fluorouracil/Leucovorin in the Adjuvant Treatment of Colon Cancer (MOSAIC) trial (7), the integration of anti-vascular endothelial growth factor(VEGF) and anti-epidermal growth factor receptor(EGFR) targeted agents (8), and, more recently, the introduction of immune checkpoint inhibitors for microsatellite instability-high (MSI-H)/deficient mismatch repair (dMMR) tumors (9). However, these gains have not been experienced equally across the population. Pronounced disparities across sex, race, and geographic location persist (10, 11).

In light of these patterns, there is a critical need for updated long-term assessments incorporating the most recent mortality data. Therefore, we undertook a nationwide retrospective analysis of CRC AAMRs from 1999 to 2023 using the Centers for Disease Control and Prevention’s Wide-ranging Online Data for Epidemiologic Research (CDC WONDER) database, with granular stratification by sex, age, race/ethnicity, state, level of urbanization, and census region. Our objective was to delineate long-term trends and the landscape of inequities, thereby generating actionable evidence for precision interventions targeting high-risk populations and high-burden areas, and informing evidence-based decision-making in colorectal oncology.

## Methods

### Study setting and population

This nationwide retrospective study used death-certificate data from the CDC WONDER platform (12). Adults aged ≥ 25 years were eligible. CRC-related deaths were identified by International Classification of Diseases, 10th Revision (ICD-10) codes C18–C20: C18 (all colonic subsites-C18.0 caecum, C18.1 appendix, C18.2 ascending colon, C18.3 hepatic flexure, C18.4 transverse colon, C18.5 splenic flexure, C18.6 descending colon, C18.7 sigmoid colon, C18.8 overlapping lesion, C18.9 colon not otherwise specified), C19 (rectosigmoid junction), and C20 (rectum). Records listing CRC as the underlying or a contributing cause of death were extracted and aggregated to calculate and compare AAMRs by sex, age, race/ethnicity, state, urban–rural classification, and census region.

Since this study used publicly available, de-identified data, institutional review board approval was not required. Reporting follows STROBE guidelines (13).

Demographic variables extracted included sex, age, calendar year, race/ethnicity, geographic region, state, and urban–rural status. Race/ethnicity was categorized by CDC WONDER as non-Hispanic White, non-Hispanic Black or African American, non-Hispanic American Indian/Alaska Native, non-Hispanic Asian/Pacific Islander, and Hispanic. Age was grouped in deciles (25–34, 35–44, 45–54, 55–64, 65–74, 75–84, and ≥ 85 years). Urbanization followed the National Center for Health Statistics (NCHS) Urban–Rural Classification Scheme, and census regions were Northeast, Midwest, South, and West (14).

### Statistical analysis

We evaluated temporal trends in CRC mortality from 1999 to 2023 and disparities across demographic subgroups (race/ethnicity, geographic region, urban–rural status). Mortality indicators included crude mortality (per 100,000 population) and AAMR (15). Crude rates were calculated as CRC deaths divided by the corresponding population. AAMRs were directly standardized to the 2000 US standard population to ensure comparability across groups with different age structures. Considering that CDC WONDER does not allow further age adjustment within specific age strata, age-stratified analyses used crude rates, which remain informative for describing the actual burden of death.

Temporal trends were modeled with the Joinpoint Regression Program (version 4.9.0.0; National Cancer Institute, USA) (16). The program fits a series of joined log-linear segments, automatically identifying joinpoints (trend-change points) that optimize model fit. The number and position of joinpoints were selected by Monte Carlo permutation or weighted Bayesian Information Criterion (BIC), the latter offering greater stability for complex data.

For each segment, we report the annual percent change (APC) with 95% confidence intervals (CIs); overall trends are summarized as the average annual percent change (AAPC) with 95% CIs, calculated as the weighted mean of segment-specific APCs. Statistical significance was assessed with two-sided *t*-tests, and *p* ≤ 0.05 was considered significant (denoted by an asterisk in figures and tables). To assess the potential influence of demographic aging on observed trends, we performed a Kitagawa decomposition to partition the overall change in crude mortality between 1999 and 2023 into two components: the rate effect, representing changes in age-specific mortality rates, and the composition effect, representing shifts in the population age distribution.

## Results

Between 1999 and 2023, colorectal cancer deaths in the USA declined by 5.6%, from 56,642 to 53,456. Over the same period, the AAMR fell from 32.06 to 19.57 per 100,000 population, yielding an AAPC of − 2.08% per year. Female deaths decreased by 14.4%, with the AAMR dropping to 16.45 per 100,000; male deaths rose by 3.4%, yet their AAMR still fell to 23.23 per 100,000. The South recorded a 12.9% increase in deaths and retained the highest AAMR (21.13 per 100,000) with the shallowest decline, whereas the Northeast showed a 33.8% reduction in deaths, the lowest AAMR (17.31 per 100,000), and the steepest decline. Non-Hispanic Black individuals maintained the highest AAMR (24.90 per 100,000) but achieved the fastest decline; in contrast, deaths among Hispanic and other non-Hispanic minority groups rose 1.5–1.7-fold, and their AAMR declined the least. Throughout the study period, nonmetropolitan counties consistently exhibited higher AAMRs than metropolitan counties (23.16 vs. 18.69 per 100,000) and declined more slowly. The AAMR for adults aged 25–44 years continued to rise (+ 0.45%–1.03% per year), whereas marked declines were observed in those aged ≥ 55 years (− 1.43% to − 3.20% per year) (**Table 1**).

**Table 1:** CRC deaths and AAMR in 1999 and 2023, with corresponding temporal trends.

Characteristic	Deaths			AAMR		
	1999	2023	Percent change (%)	1999 (95% CI)	2023 (95% CI)	AAPC (95% CI)
Overall	56,642	53,456	− 5.62	32.06 (31.80 to 32.33)	19.57 (19.40 to 19.74)	− 2.08 (− 2.22 to − 1.99)^*^
Sex						
Female	28,598	24,471	− 14.43	27.25 (26.93 to 27.56)	16.45 (16.24 to 16.66)	− 2.12 (− 2.31 to − 1.99)^*^
Male	28,044	28,985	3.36	38.87 (38.41 to 39.33)	23.23 (22.95 to 23.50)	− 2.15 (− 2.23 to − 2.06)^*^
Census region						
Northeast	13,025	8,622	− 33.80	35.03 (34.43 to 35.63)	17.31 (16.94 to 17.68)	− 3.08 (− 3.18 to − 2.98)^*^
Midwest	14,303	11,516	− 19.49	33.99 (33.43 to 34.55)	20.13 (19.75 to 20.50)	− 2.22 (− 2.34 to − 2.12)^*^
South	19,511	22,030	12.91	31.33 (30.89 to 31.77)	21.13 (20.84 to 21.41)	− 1.74 (− 1.89 to − 1.63)^*^
West	9,803	11,288	15.15	27.79 (27.24 to 28.34)	18.31 (17.97 to 18.65)	− 1.75 (− 1.90 to − 1.62)^*^
Race						
Hispanic	2,012	4,999	148.46	21.98 (20.97 to 22.99)	16.08 (15.62 to 16.54)	− 1.33 (− 1.51 to − 1.19)^*^
NH Black	6,643	6,993	5.27	44.00 (42.93 to 45.07)	24.90 (24.30 to 25.50)	− 2.49 (− 2.69 to − 2.35)^*^
NH White	46,855	38,649	− 17.51	31.72 (31.44 to 32.01)	20.04 (19.83 to 20.24)	− 1.94 (− 2.07 to − 1.85)^*^
NH Other	985	2,687	172.79	19.15 (17.88 to 20.42)	13.56 (13.04 to 14.08)	− 1.76 (− 1.96 to − 1.52)^*^
Urbanization^#^						
Metropolitan	45,617	43,353	− 4.96	31.79 (31.50 to 32.09)	18.69 (18.50 to 18.87)	− 2.56 (− 2.66 to − 2.46)^*^
Nonmetropolitan	11,025	10,144	− 7.99	33.25 (32.63 to 33.87)	23.16 (22.69 to 23.64)	− 1.81 (− 1.94 to − 1.65)^*^
Age^##^						
25–34 years	277	367	32.49	0.69 (0.69 to 0.69)	0.81 (0.81 to 0.81)	0.45 (0.10 to 0.81)^*^
35–44 years	1,295	1,645	27.03	2.87 (2.87 to 2.87)	3.71 (3.71 to 3.71)	1.03 (0.85 to 1.20)^*^
45–54 years	3,981	4,916	23.49	10.88 (10.88 to 10.88)	12.14 (12.14 to 12.14)	0.45 − 0.22 to 1.11)
55–64 years	7,886	10,153	28.75	33.17 (33.17 to 33.17)	24.26 (24.26 to 24.26)	− 1.43 (− 1.72 to − 1.13)^*^
65–74 years	14,147	13,819	− 2.32	76.81 (76.81 to 76.81)	39.84 (39.84 to 39.84)	− 2.69 (−2.97 to − 2.40)^*^
75–84 years	17,829	12,789	− 28.27	145.84 (145.84 to 145.84)	69.63 (69.63 to 69.63)	− 3.20 (− 3.29 to − 3.11)^*^
85+ years	11,227	9,767	− 13.00	270.27 (270.27 to 270.27)	157.66 (157.66 to 157.66)	− 2.35 (− 2.68 to − 2.02)^*^

For the 2023 urbanization categories, AAMR values are based on 2020 data, and the AAPC is calculated for the full period from 1999 to 2023. For the age-group analysis, AAMR represents the crude mortality rate, and the corresponding AAPC is derived from these crude rates. All *p*-values are two-sided; *p* < 0.05 was considered statistically significant.

CRC, colorectal cancer; AAMR, age-adjusted mortality rate; CI: 95%, confidence interval; AAPC, average annual percent change.

^*^AAPCs that achieve statistical significance.

^#^ For the 2023 urbanization categories, AAMR values are based on 2020 data, and the AAPC is calculated for the full period from 1999 to 2023.

^##^ For the age-group analysis, AAMR represents the crude mortality rate, and the corresponding AAPC is derived from these crude rates.

### Gender

**Figure 1:**
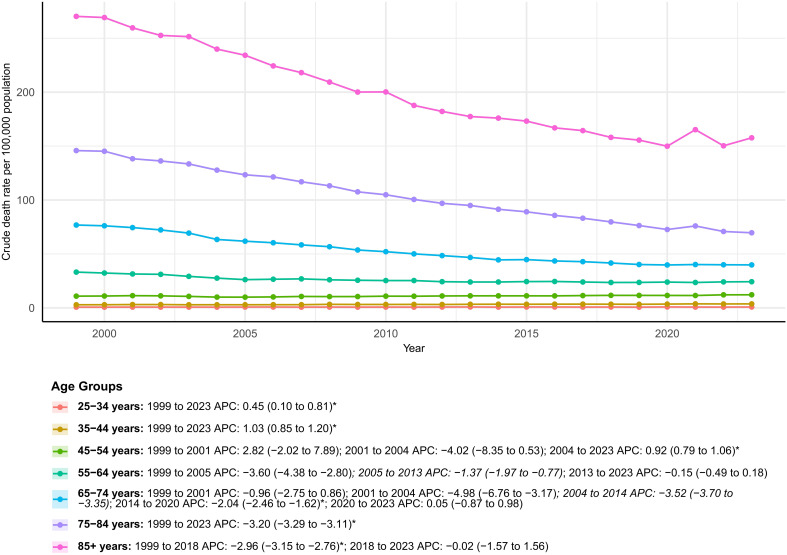
Temporal trends in age-adjusted colorectal cancer mortality by age group, USA, 1999–2023. Trends in AAMRs across age groups from 1999 to 2023, with Joinpoint-derived APC values indicating periods of increase or decline. * indicates statistically significant APC different from 0 at α = 0.05.

Deaths in the 25–34- and 35–44-year groups increased by 32.5% and 27.0%, respectively, with AAMRs rising from 0.69 to 0.81 and 2.87 to 3.71 per 100,000 population; their AAPCs were + 0.45%/year (*p* = 0.015) and + 1.03%/year (*p* < 0.001), indicating an upward shift in mortality risk among young adults. By contrast, AAMRs fell significantly in the 55–64, 65–74, 75–84, and ≥ 85-year groups (AAPCs − 1.43%, − 2.69%, − 3.20%, and − 2.35% per year, all *p* < 0.001). Although rates declined, individuals aged ≥ 75 years still accounted for 52.6% of deaths across the study period, including 268,119 deaths among those ≥ 85 years, underscoring a substantial absolute burden (**Supplementary Table S1**; **Figure 1**).

### Census regions

**Figure 2:**
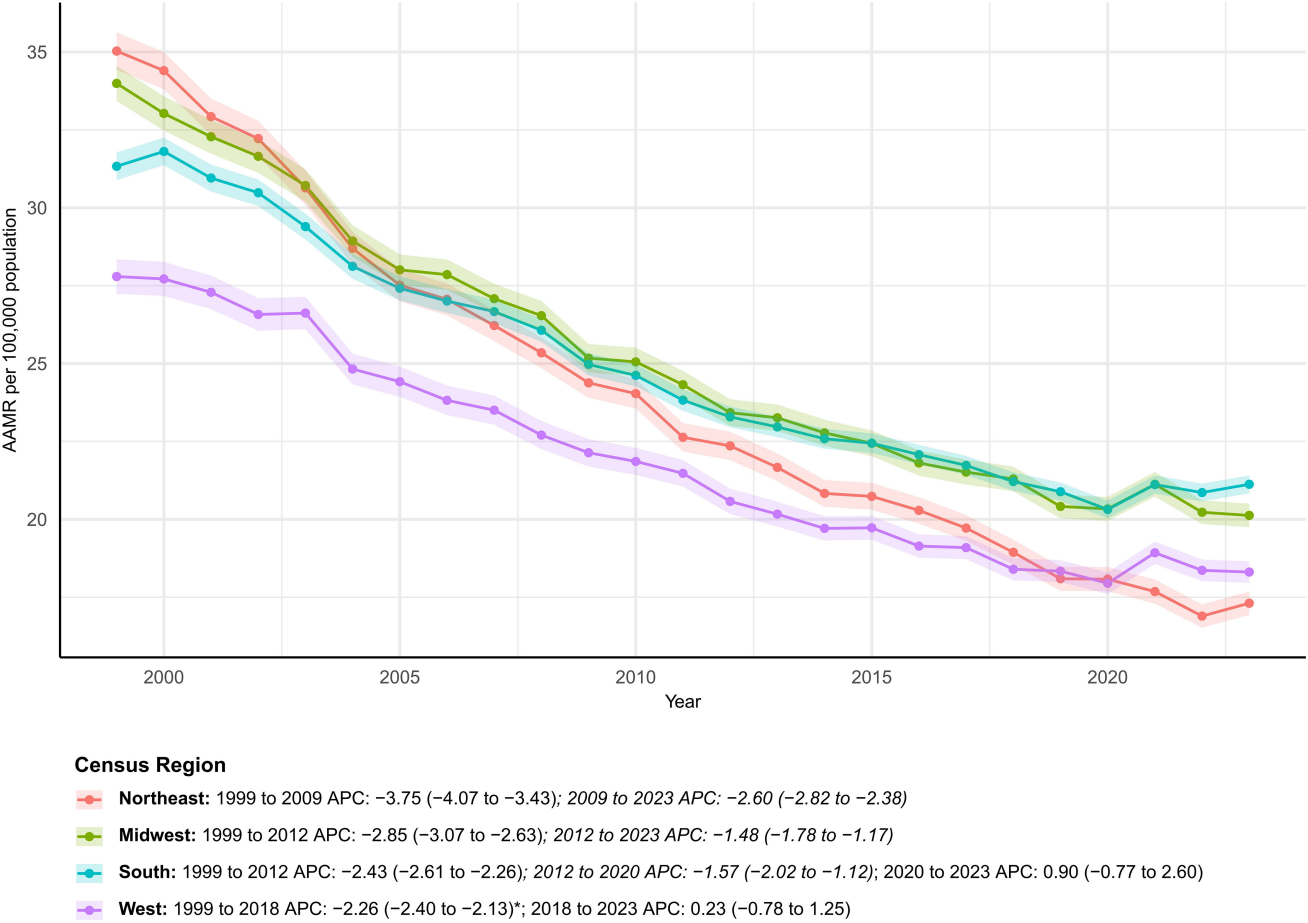
Temporal trends in age-adjusted colorectal cancer mortality by census region, USA, 1999–2023. AAMR trends across the Northeast, Midwest, South, and West, showing regional differences in the pace and timing of mortality decline. * indicates statistically significant APC different from 0 at α = 0.05.

From 1999 to 2023, only the South (+ 12.9%) and West (+ 15.1%) experienced net increases in deaths, whereas the Northeast and Midwest recorded decreases of 33.8% and 19.5%. In 2023, a regional mortality gradient persisted: South, 21.13; Midwest, 20.13; West, 18.31; and Northeast, 17.31 per 100,000 population. Trend analysis showed the Northeast declining fastest (AAPC − 3.08%/year) and the South slowest (− 1.74%/year). Cumulative burden was greatest in the South (39.6%) (**Supplementary Table S2**; **Figure 2**).

### State

**Figure 3:**
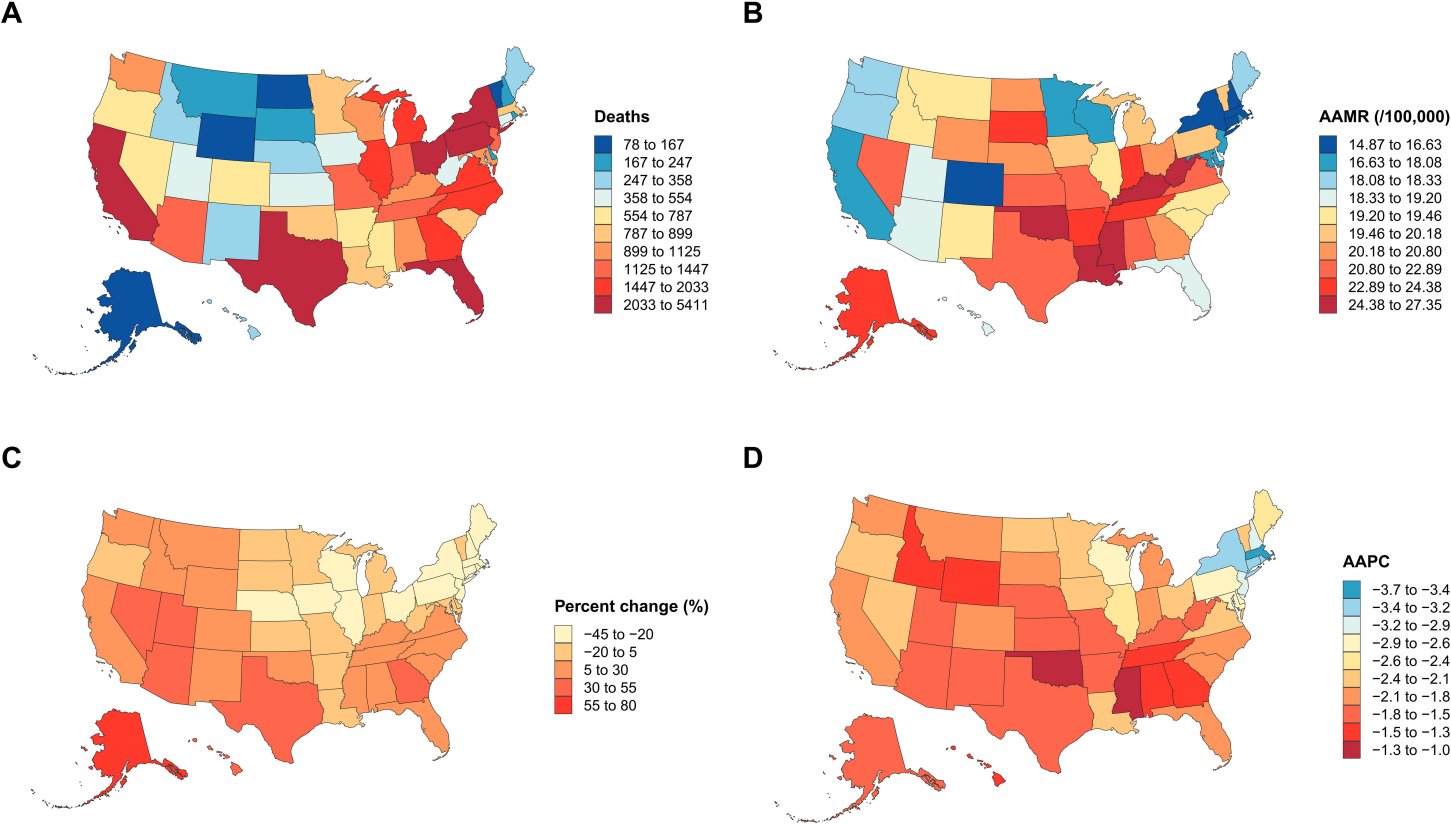
State-level disparities and temporal trends in colorectal cancer mortality in the USA, 1999–2023. **(A)** Total number of CRC deaths by state during 1999–2023. **(B)** Mean age-adjusted mortality rate (AAMR, per 100,000 population) by state for the same period. **(C)** Percent change in AAMR between 1999 and 2023, showing the magnitude and direction of temporal shifts in mortality. **(D)** Average annual percent change (AAPC) in AAMR derived from Joinpoint regression, indicating state-specific mortality trends over time.

The five states with the highest cumulative deaths were California, Florida, Texas, New York, and Pennsylvania, accounting for 37.8% of the national total. In 2023, the highest AAMRs clustered in the South–Appalachia corridor—Mississippi, Kentucky, Oklahoma, West Virginia, and Louisiana (24.43–27.34 per 100,000)—whereas the lowest were in the Northeast/Rocky Mountain region—Massachusetts, Connecticut, New York, New Hampshire, and Colorado (14.88–16.63 per 100,000). The steepest declines occurred in Massachusetts and the District of Columbia (to − 3.61%/year) and the slowest in Oklahoma and Mississippi (to − 1.13%/year) (**Supplementary Table S3**; **Figure 3**).

### Race

**Figure 4:**
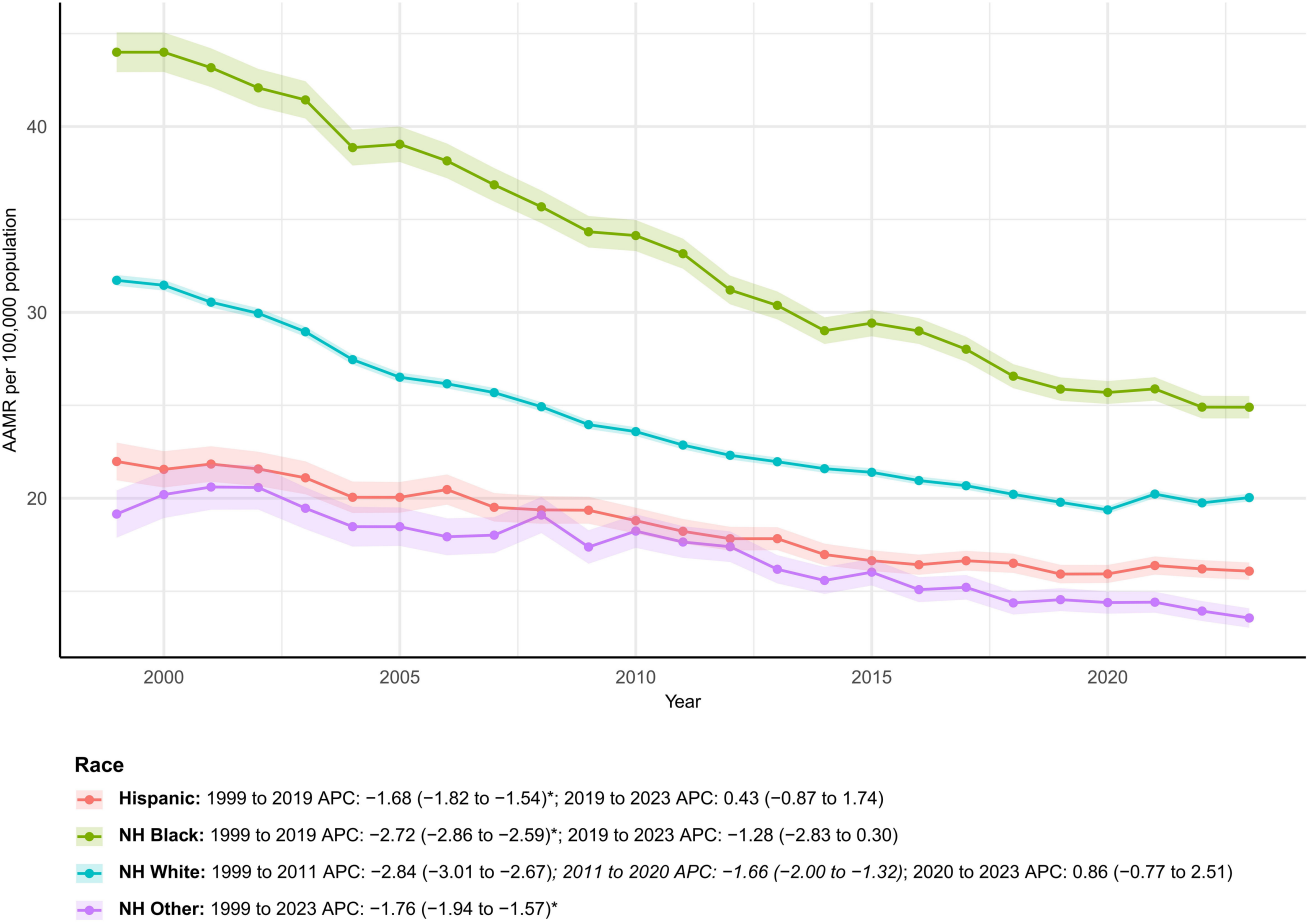
Temporal trends in age-adjusted colorectal cancer mortality by race, USA, 1999–2023. Long-term AAMR trends across racial and ethnic groups, highlighting persistent disparities. * indicates statistically significant APC different from 0 at α = 0.05.

Non-Hispanic Whites (NH White) remained the largest contributor to deaths (1,022,785 cumulative; annual deaths − 17.5%), yet Non-Hispanic Blacks (NH Black) sustained the highest AAMRs throughout the period (1999: 44.0; 2023: 24.9 per 100,000) despite a more rapid decline (AAPC − 2.49%/year). Hispanics and “Other” non-Hispanic minorities showed the largest increases in deaths (+ 148.5% and + 172.8%), and their AAMR declines were the slowest among the four groups (− 1.33%/year and − 1.76%/year, respectively). Although NH White and NH Black combined accounted for 87.6% of deaths, the share attributable to Hispanics and NH Other rose from 7.1% to 17.3%, reflecting an accelerating burden among minority populations (**Supplementary Table S4**; **Figure 4**).

### Sex

**Figure 5:**
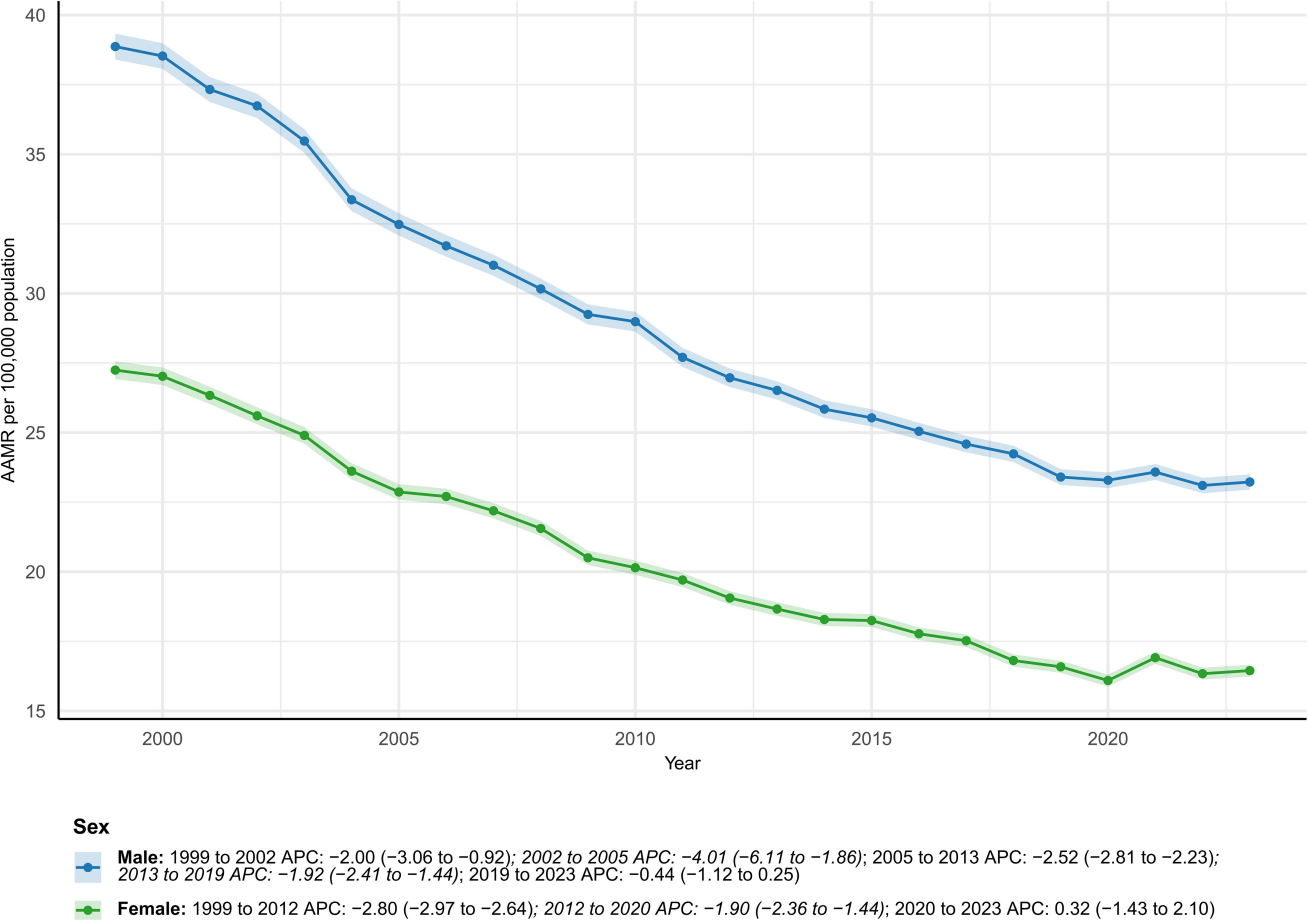
Temporal trends in age-adjusted colorectal cancer mortality by sex, USA, 1999–2023. Declining AAMRs for both sexes, with consistently higher mortality among men. * indicates statistically significant APC different from 0 at α = 0.05.

Between 1999 and 2023, there were 685,793 male and 637,816 female deaths. Male deaths rose by 3.4%, while female deaths fell by 14.4%, yielding an overall decline of 5.6%. AAMRs fell markedly in both sexes (male deaths 38.87 to 23.23; female deaths 27.25 to 16.45 per 100,000), yet the male excess in 2023 remained 6.78 per 100,000. AAPCs were similar (male deaths − 2.15%; female deaths − 2.12%) (**Supplementary Table S5**; **Figure 5**).

### Urbanization

**Figure 6:**
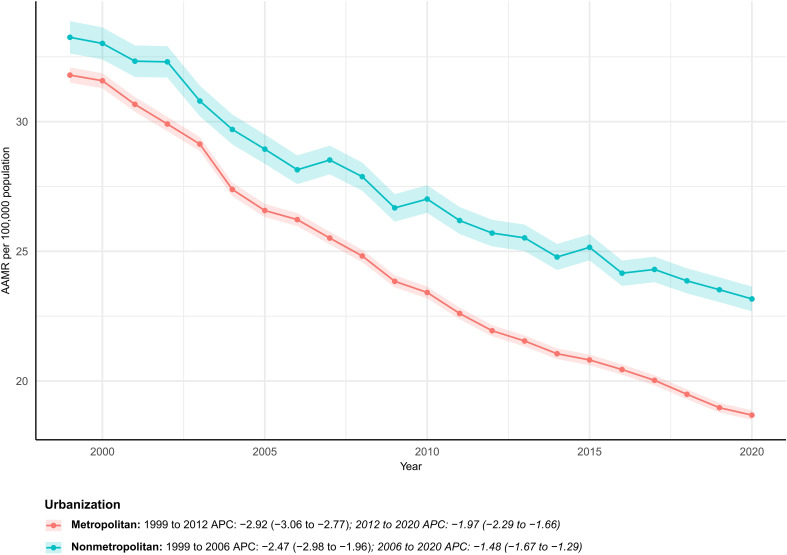
Temporal trends in age-adjusted colorectal cancer mortality by urbanization, USA, 1999–2023. AAMR trends for metropolitan and nonmetropolitan populations show persistent rural disadvantage. * indicates statistically significant APC different from 0 at α = 0.05.

Metropolitan counties accounted for 80.6% of total deaths. However, nonmetropolitan counties had a higher baseline AAMR (1999: 33.25 vs. 31.79 per 100,000) and declined more slowly (AAPC 1.81% vs. 2.56%), reinforcing a persistent urban–rural mortality gap (**Supplementary Table S6**; **Figure 6**).

### Decomposition of population structure versus rate change (1999–2023)

From 1999 to 2023, the crude mortality rate of colorectal cancer in the USA declined by 8.3 per 100,000. Kitagawa decomposition showed that decreases in age-specific mortality contributed − 14.0 per 100,000, while population aging offset this decline by + 5.7 per 100,000. Overall, the mortality reduction was mainly driven by risk decreases, partly attenuated by demographic aging.

## Discussion

Using nationwide death-certificate data from CDC WONDER (1999–2023), this study delineated long-term trends and heterogeneity in US CRC mortality. We documented an upward shift in mortality risk among young adults aged 25–44 years, whereas older adults experienced a sustained and steeper decline. The South maintained a high-burden-but-slow-decline pattern that contrasted with the Northeast’s high-baseline-but-rapid-decline trajectory. Racial/ethnic analyses showed that NH Black individuals continued to exhibit the highest mortality rates yet achieved the fastest reduction, whereas deaths among Hispanics and “other” NH Other individuals rose steadily. Male risk declined only modestly, and nonmetropolitan counties persistently displayed higher rates with slower improvement. These findings provide robust population-level context to inform precision interventions for high-risk groups and high-burden regions (17).

Although age-specific mortality rates substantially declined between 1999 and 2023 (− 14.0 per 100,000), population aging offset this improvement by + 5.7 per 100,000. In other words, if the population age distribution had remained constant at the 1999 level, the overall decline in crude mortality would have been almost twice as large. This pattern suggests that the apparent moderation in the overall mortality decline is more consistent with structural demographic shifts in the USA than with limited progress in disease control.

The rise in AAMR among adults aged 25–44 years aligns with global data on the increasing incidence of early-onset CRC (EOCRC) (18). Shifts in risk-factor exposure and lifestyle over the past two decades—including energy surplus and the obesity epidemic, alcohol and tobacco use, physical inactivity and sleep deprivation, consumption of ultra-processed foods and sugar-sweetened beverages, and early broad-spectrum antibiotic exposure with consequent microbiome alterations (19–23)—have all been proposed as potential contributors to the heightened risk of EOCRC. Moreover, the recommended age for initiating average-risk screening was lowered from 50 to 45 years (24). Although this change may transiently elevate incidence, it is expected to be temporally aligned with, and may ultimately contribute to, reducing CRC mortality in high-risk cohorts over the long term, although definitive mortality effects will require longer follow-up.

Among older adults, the AAMR for CRC showed a pronounced downward trend that is temporally aligned with both improved screening uptake and key therapeutic advances. In colon cancer, the MOSAIC trial established FOLFOX as the adjuvant chemotherapy gold standard (7). For rectal cancer, the German CAO/ARO/AIO-94 trial demonstrated that preoperative chemoradiotherapy enhances local control while reducing toxicity (25, 26). At the metastatic stage, anti-VEGF/EGFR targeted agents such as bevacizumab and cetuximab have significantly extended overall survival, and first-line PD-1 inhibitors for MSI-H/dMMR tumors have further improved progression-free survival. Most notably, immune neoadjuvant therapy for localized dMMR rectal cancer has achieved unprecedented complete clinical-response rates (9, 27). These milestone therapeutic evolutions, in concert with early cancer screening, are temporally aligned with, and may collectively contribute to, the continuing annual decline in AAMR among middle-aged and older populations.

Male AAMRs have persistently exceeded those of female AAMRs and have declined only modestly—a pattern that may reflect behavioral differences and, potentially, sex-specific biological mechanisms that remain to be clarified. Compared with women, men more frequently engage in high-risk behaviors such as smoking, alcohol consumption, central obesity, and occupational exposures, all of which are associated with elevated CRC risk (21, 22); lower screening adherence among men further compounds the disparity. In addition, “escape from X-inactivation tumor-suppressor genes” (EXITS) may confer women a dual-allelic tumor-suppressive advantage, partly explaining men’s higher incidence of certain cancers (28). Sex chromosomes, modulated by sex hormones, also shape anticancer immunity (29), and women generally mount stronger innate and adaptive immune responses than men (30). These hormonal and immunological differences may partly underlie sex-specific variation in disease progression and overall survival, underscoring the need for tailored interventions to address men’s relatively elevated CRC mortality risk.

We observed that the NH Black population still bears the highest AAMR, yet its recent decline has accelerated—a pattern that is temporally aligned with documented improvements in colonoscopic screening. In Delaware, a 9-year initiative by the Delaware Cancer Consortium was associated with the elimination of the CRC incidence gap between NH Black (47.8% → 73.5%) and White residents through colonoscopy-based screening (31). By contrast, deaths among Hispanics and “other” minorities have risen sharply. Multiple studies demonstrate lower colonoscopy uptake in minority groups (32–35) and poorer follow-up of abnormal findings (36). Likely contributing factors include socioeconomic status, residential segregation, insurance and payment barriers, and limited access to high-quality screening and treatment (37). Pragmatic trials show that mailed FIT programs successfully boost screening in underserved populations and can narrow racial/ethnic gaps (38, 39). These findings align with our “high-burden, slow-decline” pattern in the South and nonmetropolitan counties and are consistent with the potential feasibility of such interventions.

Cancer mortality burdens are higher and decline more slowly in nonmetropolitan than metropolitan counties. Rural residents face greater public-health challenges owing to restricted healthcare access, fewer screening opportunities, and higher poverty rates (40, 41). Urban–rural gaps correlate with endoscopy capacity, follow-up colonoscopy completion rates, transportation and leave costs, health literacy, and shortages of primary-care resources (42). At the state level, AAMRs remain elevated and fall sluggishly along the South-Appalachia corridor, whereas several Northeastern states exhibit faster declines. The corridor’s relative economic disadvantage and high rurality may help explain steeper AAMR increases, while the more urban Northeast posts the nation’s lowest rates—further supporting links between CRC mortality and socioeconomic inequity.

Drawing on nationwide data spanning more than two decades, our results closely mirror the latest periodic report in *CA: A Cancer Journal for Clinicians*: overall CRC mortality continues to fall, yet the rise in early-onset CRC and persistent structural disparities remain worrisome (11). These observations are consistent with the USPSTF’s 2021 recommendation to begin average-risk screening earlier (17) and echo prior literature on racial, urban–rural, and interstate differences, underscoring the urgent need for precision interventions targeting EOCRC and health inequities.

## Limitations

This study has several limitations. First, it relied on nationwide, aggregated death-certificate data from CDC WONDER for 1999–2023, all coded using ICD-10; misclassification due to coding error remains possible. Second, the dataset lacks information on tumor stage, treatment modalities, comorbidities, insurance status, and medication use, and it does not capture indicators of healthcare accessibility. Third, urban–rural and interstate differences may be influenced by unmeasured population migration and shifting age structures, and platform constraints limited age-stratified analyses to crude rates, reducing inter-regional comparability. Fourth, we did not explicitly stratify racial disparities by the 65-year threshold, which marks Medicare eligibility; our estimates, therefore, cannot fully account for differences in insurance coverage and access to care above and below this age, and the observed racial patterns should be interpreted in this context. Finally, reporting lags restrict near–real-time assessment of the most recent mortality patterns.

## Data Availability

Publicly available datasets were analyzed in this study. This data can be found here: https://wonder.cdc.gov/.
